# Transvaginal hysterotomy for cesarean scar pregnancy in 40 consecutive cases

**DOI:** 10.1007/s10397-014-0863-3

**Published:** 2014-10-22

**Authors:** Zhang Huanxiao, Chen Shuqin, Jiang Hongye, Xie Hongzhe, Niu Gang, Xu Chengkang, Guan Xiaoming, Yao Shuzhong

**Affiliations:** 1 Department of Gynecology & Obstetrics, The First Affiliated Hospital of Sun Yat-sen University, No. 58 2nd Zhongshan Road, Guangzhou, Guangdong Province China; 2 Baylor College of Medicine, Houston, USA

**Keywords:** Cesarean scar pregnancy, Treatment, Transvaginal hysterotomy

## Abstract

To propose a novel procedure as a safe and effective treatment for cesarean scar pregnancy (CSP), a cohort study was initiated in patients diagnosed with CSP and treated with transvaginal hysterotomy from December 2009 to March 2013, either as a primary or secondary therapy. All diagnoses were confirmed by both sonography and pathology, either a gestational sac or residual tissue after termination of pregnancy or miscarriage in the cesarean section scar. Basic clinical characteristics and perioperative data were collected and analyzed. A total of 40 patients were included. The mean age was 32.88 ± 4.55 years. The mean size of gestational sacs of the CSP mass at diagnosis was 33.78 ± 13.14 mm. Mean serum β-hCG level at diagnosis was 47379.73 ± 45285.10 IU/L. Mean operative time was 57.25 ± 24.52 min. Mean postoperative hemoglobin drop was 1.635 ± 0.906 g/dL. Complications were one case of bacteremia and two cases of hematoma. Mean hospital stay after surgery was 4.95 ± 2.62 days. Mean serum β-hCG levels decreased by 88.5, 93.5, and 96.5 % at postoperative day 2, 4, and 6, respectively. All patients’ β-hCG levels returned to normal range within 1 month after surgery. Transvaginal hysterotomy with removal of ectopic pregnancy tissue and repair of cesarean scar defect is a promising approach to manage CSPs, with a short hospital stay, low postoperative pain, blood loss, and cost.

**Electronic supplementary material:**

The online version of this article (doi:10.1007/s10397-014-0863-3) contains supplementary material, which is available to authorized users.

## Introduction

As a rare form of ectopic pregnancy, cesarean scar pregnancy (CSP) refers to the implantation of a pregnancy within the myometrium at the site of a prior cesarean scar. If not detected early and managed properly, CSP can result in life-threatening complications, such as massive hemorrhage, uterine rupture, disseminated intravascular coagulation, and even maternal death [1]. Once diagnosed, therapy is strongly recommended to avoid subsequent life-threatening complications. However, no obvious most effective therapeutic strategy has been established to date. Removal of the ectopic pregnancy tissue is usually done. By following the concepts of minimally invasive surgery, we designed and carried out the first case of hysterotomy by transvaginal approach for the treatment of CSP in 2009. A preliminary report of six cases was published in 2011 with encouraging results [2]. Since 2009, we have successfully completed 40 transvaginal CSP cases without significant complications. Herein, we summarize our experience with this novel surgical approach for CSP and propose the procedure as a safe, effective, and minimally invasive treatment modality.

## Patients and methods

All patients diagnosed with CSP and consented to have a transvaginal surgery were enrolled in this study from December 2009 to March 2013. The study protocol was approved by the institutional ethics committee of the university, and all patients provided written informed consent before enrollment.

### Diagnosis and grouping

Cesarean scar pregnancy was diagnosed by ultrasonography and β-human chorionic gonadotropin (β-hCG) level. The diagnostic criteria for transvaginal ultrasound were as follows: (1) an empty uterine cavity and cervical canal with a clearly demonstrated endometrium and (2) a gestation sac, with or without fetal cardiac activity, embedded in and surrounded by the myometrium and the fibrous tissue of the cesarean section scar in the anterior part of the uterine isthmus, that was separated from the endometrial cavity or fallopian tube, with a diminished or absent myometrial layer between the bladder and the sac [3–5]. Patients with residual in the cesarean scar tissue following spontaneaous miscarriage, termination of pregnancy, or previous treatment of CSP were also included as a subgroup. These were patients in whom the diagnosis was made based on prolonged vaginal bleeding, elevated β-hCG level, and a heterogeneous mass at the same site as the gestational sac described above. Previous treatments were considered unsatisfactory if the level of β-hCG failed to decrease 1 week after treatment, or massive vaginal bleeding developed.

To confirm the diagnosis, morphologic and pathological information were collected during and after surgery.

### Treatment protocol

**Fig. 1:**
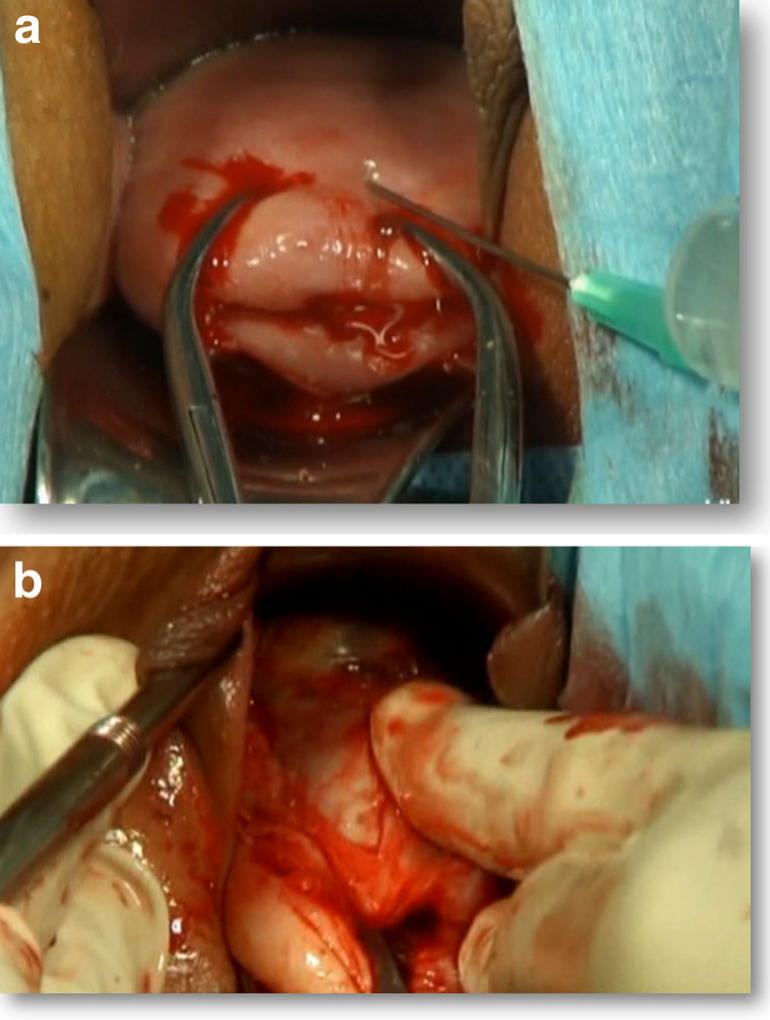
**a** Adrenaline solution was injected submucosally for hydrodissection and hemostasis. **b** The bladder was dissected away and the CSP (*arrow*) was identified as a bluish bulge located in the anterior wall of the lower uterine segment

**Fig. 2:**
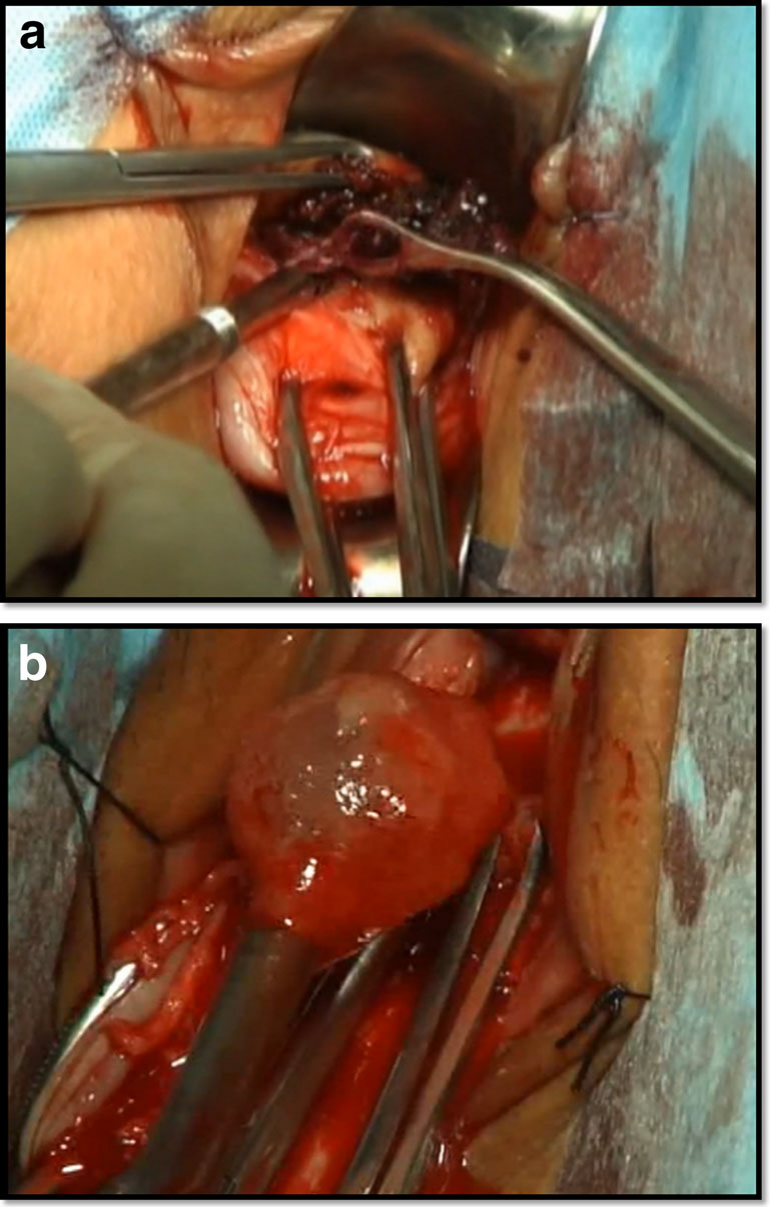
Ectopic pregnancy tissue was removed with sponge forceps (**a**) or suction (**b**) through the incision on the uterus isthmus

**Fig. 3:**
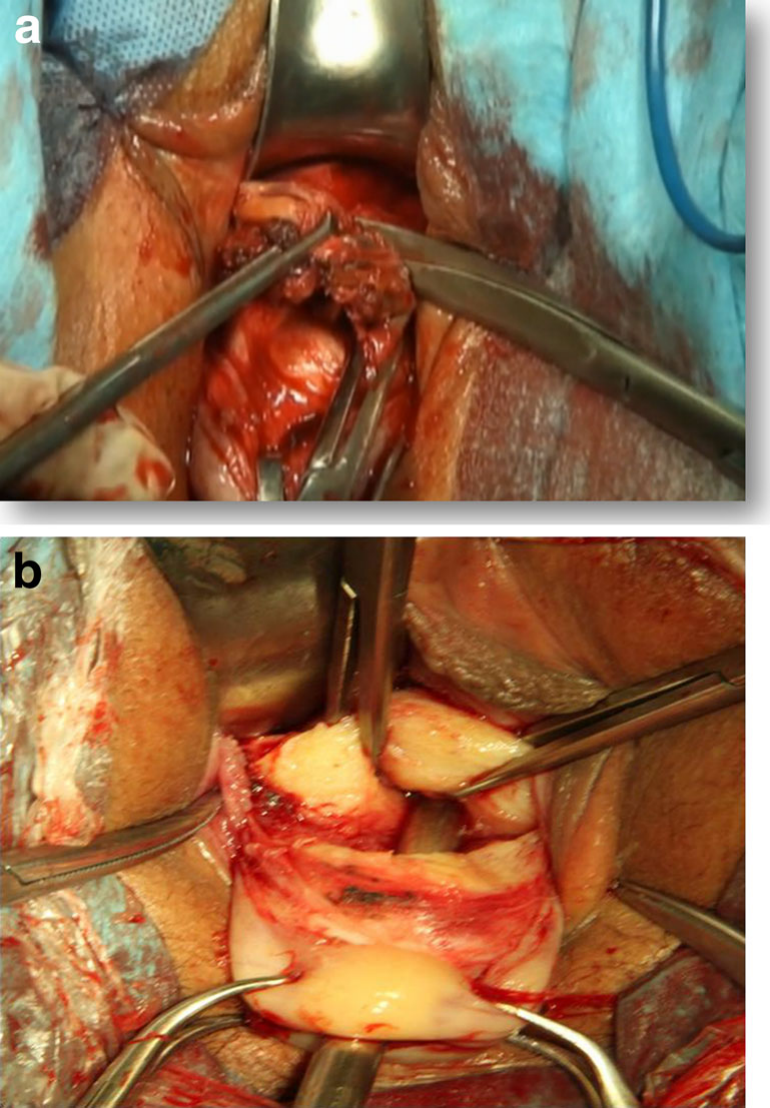
The edges of the incision were trimmed with scissors to remove all scar tissue

**Fig. 4:**
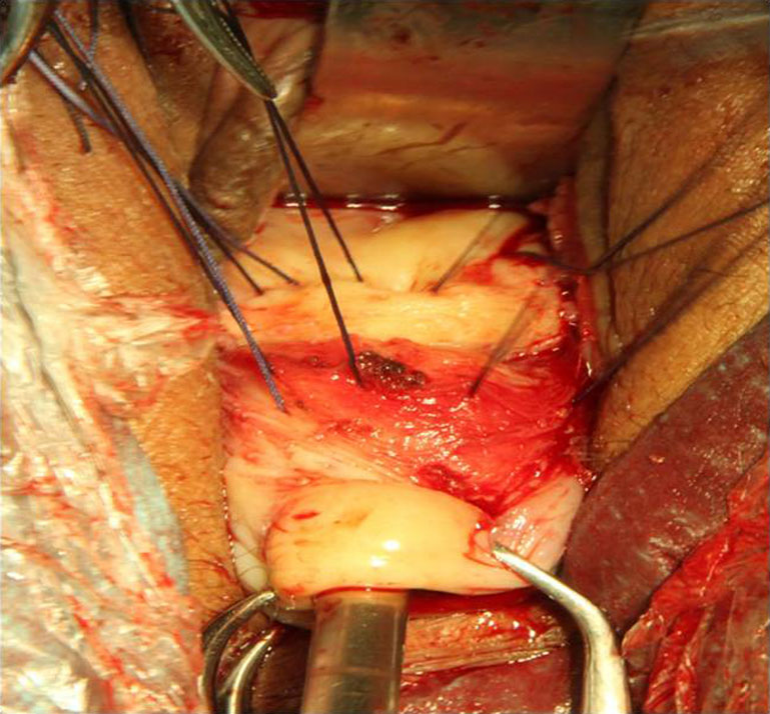
The myometrial incision was closed with interrupted suture and then continuous locking sutures

**Fig. 5:**
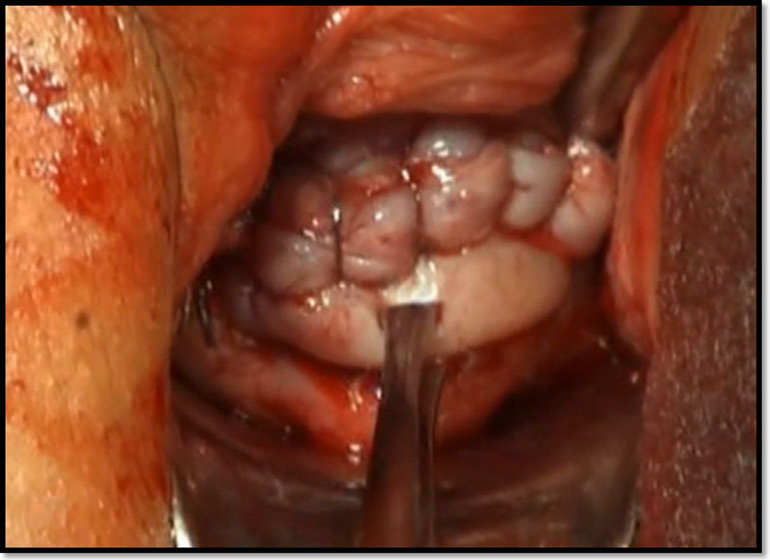
The vaginal incision was closed with continuous locking sutures

Preoperative preparation was the same as for other transvaginal surgical procedures. All patients were placed in a dorsal lithotomy position, and the bladder was emptied. General anesthesia was applied. After a pair of vaginal retractors was placed into the vagina, the cervix was grasped and manipulated with a toothed tenaculum. Adrenaline solution (1.5 μg/mL; 10–20 mL) was injected submucosally for hydrodissection and hemostasis before an incision was made at the anterior cervicovaginal junction (Fig. 1a). The bladder was dissected away until the anterior peritoneal reflection was identified. The anterior retractor was inserted into the vaginal incision to lift up the bladder. The CSP was identified as a bluish bulge located in the anterior wall of the lower uterine segment (Fig. 1b). A transverse incision was made on the lower margin of the most prominent area of the bulge. Ectopic pregnancy tissue was removed with sponge forceps or suction through the incision on the uterus isthmus (Fig. 2). Thorough curettage was done through the incision or cervical canal. The edges of the incision were trimmed with scissors to remove all of the scar tissue (Fig. 3). The myometrial (Fig. 4) and vaginal (Fig. 5) incisions were closed with continuous locking sutures using 2–0 absorbable sutures.

### Postoperative assessment

Perioperative parameters included operating time, blood loss, blood transfusion, conversion to laparotomy, complications related to surgery, course of serum β-hCG titers, length of hospital stay after surgery, and other adverse effects.

Follow-up assessments included remission of symptoms, monitoring decrease of serum β-hCG titer, and resumption of menstruation thereafter. Serum β-hCG level was monitored every other day in the first week after the surgery and weekly thereafter until the titers returned to normal levels.

### Statistical analysis

Data were collected and evaluated for parametric analysis. Mean ± standard deviations (SD) are reported for continuous data, whereas categorical data are presented as the absolute count and percentage. ANOVA test was used to compare the mean differences. *P* < 0.05 was considered statistically significant. SPSS 13.0 software was used to perform statistical analysis.

## Results

A total of 40 consecutive patients with CSP who underwent vaginal surgery were enrolled in the study. The baseline clinical characteristics are presented in Table 1. Twenty-five patients presented with vital pregnancy (Group A), 23 of which had fetal heart activity at the moment of surgery. Eight patients underwent medical treatment or embolization prior to admission (Group B), including two cases of uterine artery embolization (UAE) combined with local methotrexate (MTX) injection, three cases of systemic MTX injection, and three cases who received mifepristone treatment. All of these were considered unsatisfactory. The other seven patients presented with residual products of conception in the cesarean scar on ultrasound scan after spontaneous or elective abortion (Group C). One patient had undergone three previous lower segment transverse cesarean sections. Fourteen patients had two previous cesarean sections. The remaining 25 patients had one previous cesarean section. Twelve patients were asymptomatic. Twenty-eight patients complained of various patterns of vaginal bleeding, including one case of hemorrhagic shock.

**Table 1:** The baseline clinical characteristics of 40 patients ^a^

Characteristic	Mean ± SD (range)^a^
Age (yr)	32.88 ± 4.55 (24–43)
Interval from last cesarean section to surgical treatment (month)	68.61±45.34 (9–188)
Gestational age (day)	58.59 ± 16.48 (41–120)
Serum β-hCG level (IU/L)	47,379.73 ± 45,285.10 (9.2–188,942)
Diameter of gestational sac (mass) on ultrasound scan (mm)	33.78 ± 13.14 (12–60)

^a^Data are given as mean ± SD (range)

Perioperative data on vaginal surgery is presented in Table 2. None of the patients required conversion to laparotomy. Blood transfusion was needed only for the single patient who presented with hypovolemic shock at admission. No excessive bleeding or injury to adjacent organ was observed. Only one patient developed postoperative gram negative septicemia as demonstrated by hemoculture. The patient completed a 7-day course of antibiotic treatment and was subsequently discharged without further signs of infection. Two patients developed a local hematoma that resolved spontaneously after 3 months with no further complications. The mean serum β-hCG level decreased by 88.5, 93.5, and 96.5 % at postoperative day 2, 4, and 6, respectively, comparing to baseline. In all patients, trophoblast and scar tissue within the uterine smooth muscle confirmed the pathologic diagnosis of CSP.

**Table 2:** Perioperative evaluation of the surgical procedure

Variable	Mean ± SD (range)^a^
Duration of surgery, (min)	57.25 ± 24.52 (20–120)
Estimated blood loss during surgery, (mL)	47.88 ± 35.71 (10–200)
Length of hospital stay after surgery, (day)	4.95 ± 2.62 (2–17)
Hemoglobulin decreased after 24 h, (g/L)	16.35 ± 9.06 (2–41)

^a^Data are given as Mean ± SD (range)

Gestational age, β-hCG level, and size of gestational sac were not associated with operating time, hemoglobin decrement, or length of hospital stay after surgery. The subgroup of patients who underwent other treatment for CSP before vaginal surgery had significantly longer operating time than those without previous treatment (73.1 ± 28.2 vs. 52.2 ± 23.2). Hemoglobin drop and length of hospital stay after surgery were not significantly different among the subgroups (Table 3).

**Table 3:** Comparison of subgroups^b^

	Sub-group^a^		
	Group A (vital CSPs without previous treatment)	Group B (CSPs with previous conservative treatment)	Group C (CSPs with pregnant tissue residues)
No. of cases	25	8	7
Age, (yr)	33.6 ± 4.4	31.0 ± 4.8	32.4 ± 4.7
Serum β-hCG level (IU/L)	58,160.4 ± 46,189.4^d^	40,481.4 ± 36067.2	16,761.0 ± 40,457.9^b^
Size of gestational sac (mass) on ultrasound scan before surgery, (mm)	34.8 ± 10.9	28.0 ± 14.2	38.0 ± 20.5
Duration of surgery, (min)	52.2 ± 23.2^c^	73.1 ± 28.2^b^	57.1 ± 19.5
Hemoglobulin decrement within 24 h, (g/L)	17.0 ± 10.1	13.3 ± 7.4	17.7 ± 6.7
Length of hospital stay after surgery, (day)	5.48 ± 3.0	4.13 ± 1.2	4.0 ± 1.7

^a^Data are given as mean (SD). The mean difference is significant at the 0.05 level

^b^Significantly different from Group A

^c^Significantly different from Group B

^d^Significantly different from Group C

Finally, patients were categorized into two cohorts of 20 by chronological order. Their age, gestational age, serum β-hCG level, and size of gestational sac were comparable. Hospital stay after surgery significantly decreased in the second group (6.0 ± 3.20 vs. 3.90 ± 1.25). Though there was a trend for shorter operating time (60.0 ± 20.33 vs. 54.5 ± 28.37) and hemoglobin drop (18.25 ± 8.69 vs. 14.45 ± 9.23), this was not significant.

Follow-up data were obtained from all patients between 2 and 36 months after surgery. Vaginal bleeding stopped around 1 week after transvaginal surgery. The serum β-hCG level in all the cases resolved to normal range within 1 month after surgery. All patients recovered without complications, no subsequent treatment was needed, and there was no further report of abnormal menstrual pattern.

## Discussion

As CSP is an iatrogenic complication of cesarean section, the incidence of CSP is likely to rise dramatically in the near future due to the increase of cesarean section rates as well as the increased utilization of diagnostic ultrasound. Since first being reported by Larsen and Solomon in 1978 [6], the number of cases of CSP showed an exponential increase. Between 1978 and 2001, only 18 cases of CSP were reported in the English literature [3]. In a later review in 2007 [4], 161 cases of CSP from 58 citations were included. Jurkovic et al. and Seow et al. have estimated the prevalence of CSP in their local population attending the early pregnancy assessment unit to be 1:1800 [7] and 1:2216 [8], respectively.

As a tertiary center in southern China, we have been dealing with CSP for a long time. Our data through 2011 showed that CSPs constituted 2.2 % of ectopic gestations in our hospital [2]. Before 2009, most of CSPs were managed by UAE and MTX administration. Since the first transvaginal hysterotomy in 2009, this treatment was explained to every patient diagnosed with CSP as well as UAE. All patients agreed to have vaginal surgery and provided informed consent. It had been successfully performed in 40 cases of CSP.

In patients with prior cesarean section, transvaginal hysterotomy through the vesicouterine fold has met conceptual hurdles mainly because of concerns for adhesions from prior cesarean section scar and for injury of the bladder during surgery. We have treated patients with β-hCG level as high as 188,942 U/mL, gestational sac as large as 60 mm in diameter, or gestational age as advanced as 10 weeks. The mean operating time was no more than 1 h, and the mean decrease in hemoglobin within 24 h was 1.635 g/dL, which is tolerable for most patients. No major complications such as massive bleeding or bladder injury were reported for any of the cases. Mean hospital stay after surgery was less than 5 days, which could be further reduced to 3 or 4 days without compromising safety. Serum β-hCG titers decreased by more than 90 % before postoperative day 4 and resolved to normal range within 1 month after surgery.

Based on our observations that prior conservative treatment did not reduce operating time or hemoglobin loss, we do not recommend prior medical treatment or UAE to improve safety or efficacy of transvaginal surgery. There was both a difference in operating time or hemoglobin drop between vital pregnancy and those with residual tissue. Although in this series there was no relationship between gestational age, β-hCG level and size of gestational sac, and safety of surgery, it is probable that it is more likely to encounter difficulties in more advanced gestation. Therefore, it would seem logical to recommend early diagnosis by transvaginal ultrasound in pregnant women with previous cesarean section, though our data cannot contribute to the risk/benefit analysis of such strategy.

Although the exact pathogenetic mechanism of CSP is unknown, a potential predisposing factor is a microtubular tract between the cesarean section scar and the endometrial canal. [3]. By removing the cesarean scar together with the pregnancy tissue, we tried to avoid such persistent canal, hence may reduce recurrence—though this cannot be proved by the current study.

Before transvaginal surgery was described, MTX administered either systemically or locally with UAE was the primary management method in our hospital. Lian et al. [15] published a series of 21 patients who received MTX (2005–2009). All patients received systemic MTX (50 mg/m^2^) as initial therapy. Only nine showed a rapid decrease of serum b-hCG within a week, while MTX failed in the remaining 12. They had additional UAE combined with local MTX. Although eventually all patients responded to MTX treatment with or without UAE, it took 2 months to achieve serum β-hCG resolution and 6 months for the masses to disappear on ultrasound. Moderate elevation of liver enzymes was documented in three patients receiving systemic MTX, low grade fever (<38 °C) in five, and low abdominal pain in three with UAE combined with local MTX. In summary, MTX administration was associated with slower recovery and more adverse effects.

Based on our experience, we suggest the following for transvaginal hysterotomy approach to CSP management. Cervical injection of diluted adrenaline solution is performed before dissecting the vesicocervical gap to reduce bleeding. This step may also help avoid injuring the bladder and urethra and prevent the occurrence of vesicovaginal fistula formation. We used suction and curettage for complete removal of the products of conception as completely as possible. Continuous yet locking sutures were used to repair the incision of the anterior uterine wall under the guidance of a probe.

Prompt hysterectomy may be warranted only for dramatic cases presenting with massive bleeding or shock. Most other therapeutic options for CSPs have been adapted from protocols used for the treatment of other locations of ectopic pregnancies. The medical therapies include systemic and local administration of methotrexate (MTX), KCl, or the combination of these agents. The surgical treatments include dilatation and curettage, selective UAE, operative hysteroscopy, laparoscopy, and laparotomy. In cases of CSPs with decreasing serial serum β-hCG levels, expectant management may be offered. However, some reports showed that expectant management was associated with a high risk of uterine rupture and, therefore, could not be recommended in most cases [8–10]. Despite the effectiveness of MTX in decreasing the serum β-hCG levels, systemic or local injection of MTX requires close monitoring for a longer period of time. MTX sometimes requires repeat injection that can be associated with chemotherapy-induced side effects, slowly decreasing serum β-hCG levels and longer hospitalization [4, 11]. Moreover, there is often incomplete reabsorption of the gestational sac with ongoing irregular vaginal bleeding. Many of these women need additional and sometimes more aggressive methods to surgically remove the persistent gestational sac and resolve the bleeding [11–13]. Uterine artery embolization has been shown to carry risks of postoperative fever and abdominal pain [14], as well as being associated with longer duration of close monitoring and hospitalization [15].

Surgery has the advantage of offering the possibility of immediate remission. Hysteroscopy can be used to observe the distribution of blood vessels at the implantation site and to separate the gestational sac from the uterine wall directly. However, these procedures require good control of hysteroscopic instruments, excellent orientation within the uterine cavity, and clear visualization, which may not always be achievable [16]. In addition, hysteroscopic resection carries a risk for bladder injury, including delayed bladder perforation by thermal injury [17]. According to Fylstra’s review [3], termination of the pregnancy with repair of the accompanying uterine scar dehiscence is probably the best treatment for the cesarean scar pregnancy. Laparoscopy is a minimally invasive option for resection of the scar, with preservation of the woman’s reproductive capacity. Although laparoscopic and laparotomy approaches have already been successfully applied in CSPs [18, 19], a case report of CSP showed that the lower edge of the uterine defect was not accessible abdominally during emergency laparotomy. An additional vaginal approach was needed to achieve hemostasis [20]. This observation supports the feasibility of transvaginal repair of the uterine defect after cesarean section. Furthermore, compared to laparoscopy, transvaginal hysterotomy has the advantage of avoiding trocar insertion, therefore reducing the risks associated to this modality [21]. In addition, transvaginal hysterotomy may decrease cost associated with the use of equipment. As transvaginal hysterotomy requires only conventional equipment and the general skills of vaginal surgery, it is more convenient and more feasible to be conducted in most hospitals at lower cost than other surgical approaches. Since our first description, several other institutions employed this protocol successfully [22].

## Conclusion

In summary, this transvaginal hysterotomy approach allows for removal of ectopic pregnancy tissue and repair of uterine defect. It is safe and effective, associated with a short hospital stay, low postoperative pain, blood loss, and cost.

## Electronic supplementary material

Below is the link to the electronic supplementary material.

ESM 1 (MPG 218,026 kb)
